# Parental Impressions and Perspectives of Efficacy in Prenatal Counseling for Single Ventricle Congenital Heart Disease

**DOI:** 10.1007/s00246-023-03355-y

**Published:** 2023-12-19

**Authors:** Taylor Hartzel Houlihan, Jill Combs, Elizabeth Smith, Elizabeth Coulter, Lucia Figueroa, Christine Falkensammer, Jill Savla, Elizabeth Goldmuntz, Karl Degenhardt, Anita Szwast, Amanda Shillingford, Jack Rychik

**Affiliations:** Fetal Heart Program, Cardiac Center at the Children’s Hospital of Philadelphia, Department of Pediatrics, Perelman School of Medicine, University of Pennsylvania, 3401 Civic Center Boulevard, Philadelphia, PA 19104 USA

**Keywords:** Prenatal counseling, Single ventricle congenital heart disease, Fetal heart disease

## Abstract

Although commonly performed, optimal techniques, strategies, and content to achieve the most effective prenatal counseling have not been explored. We investigate the efficacy of prenatal counseling via survey feedback of parents of children with prenatally diagnosed single ventricle. Grades of counseling using a Likert scale (1–5) were solicited to assess: (1) overall impression of quantity of counseling, (2) explanation of the heart defect, (3) preparation for heart surgery, (4) preparation for hospital course and care, (5) preparation for complications and outcomes of a Fontan circulation, and (6) preparation for neurological, school-related, or behavioral problems. Impressions were solicited concerning specific providers. A comprehensive fetal counseling score was calculated for each participant. Burden of care including length of hospitalization was explored as impacting prenatal counseling grades. There were 59 survey respondents. Average age of the children at the time of survey was 4.6 ± 3.3 years (range 1–10 years). Highest grades were for explanation of the heart condition, with lowest grades for preparation for neurological, school-related, or behavioral problems. Cardiac surgeon received the highest with social worker lowest grade for provider. Negative correlation was found between the composite fetal counseling score and parental recollection of length of hospitalization (Pearson *r* = − 0.357, *p* < 0.01). Prenatal counseling for neurological, school-related, and behavioral problems in single ventricle is deficient. Further studies analyzing prenatal counseling techniques and content can help improve upon the delivery of this important aspect of prenatal care.

## Introduction

Obstetrical ultrasound and fetal echocardiography readily allow for the identification of a wide variety of congenital heart anomalies before birth. Prenatal detection of congenital heart disease provides the opportunity for proper education of prospective parents. Knowledge concerning impact on the pregnancy, obstetrical and delivery plans, extent of need for surgical intervention, and survival outcomes are domains of information to explore and proficiently convey in conversation with prospective parents, a process known as prenatal counseling [1]. Good counseling accompanies good diagnostics when delivering optimal prenatal care [2].

A major challenge and impediment to effective prenatal counseling is the traumatic shock to unsuspecting families following detection of congenital heart disease. Capacities for learning and processing of information are limited following receipt of traumatic news. Prenatal counseling involves conveying a wealth of complex information while the emotional and psychological state of expectant parents is impaired [3]. Although such counseling is commonly performed, optimal styles, strategies, techniques and content to achieve the most effective prenatal counseling for congenital heart disease have not been explored.

One condition in which there is great need for detailed comprehensive prenatal counseling is following fetal diagnosis of single ventricle type of congenital heart disease (SVCHD). Treatment demands a series of complex surgical interventions, however, there is no cure. Outcomes continue to improve, with most fetuses diagnosed with SVCHD today likely to survive through the stages of surgical care [4], however, quality and duration of life is variably limited. Furthermore, the condition is associated with extra-cardiac challenges including neurodevelopmental deficits and psychological challenges, as well as a host of other organ system deficits with continuing needs for life-long care [5]. Conveying to expectant parents a comprehensive understanding of SVCHD in a sensitive, compassionate, and effective manner can be a challenge [6].

Little research has been undertaken to gauge the efficacy of prenatal counseling for SVCHD. Such investigation could be of value in providing important feedback to providers and identify specific areas for improvement. In this study, we explore the efficacy of our prenatal counseling via feedback obtained from a survey administered to parents of children prenatally diagnosed with SVCHD. We report on the parental grades of prenatal counseling in various domains of information and by various providers, as well as explore variables of bias such as burden of disease that may influence perceptions of prenatal counseling for this complex cardiac malformation.

## Methods

A retrospective chart review was performed to identify families with prenatal diagnosis of single ventricle congenital heart disease cared for at the Fetal Heart Program (FHP) at Children’s Hospital of Philadelphia (CHOP) for a 10-year period (2010–2020). Candidates were those who had bidirectional Glenn and were scheduled to shortly undergo, or had already undergone, Fontan operation. Institutional review board permission for the study was obtained (CHOP IRB 20-018209). Excluded from this cohort are families that declined surgical intervention, underwent heart transplant, or incurred death prior to Fontan procedure. All subjects were managed and counseled prior to onset of COVID-19 restrictions, as pandemic protocols temporarily, but substantially, altered practice.

### Fetal Heart Program Method of Counseling

Prenatal counseling at the Fetal Heart Program at Children’s Hospital of Philadelphia (CHOP) is offered by a dedicated group of providers including a core team of experienced fetal cardiologists, fetal heart nurse coordinators, and fetal heart social worker. Although some individualized variability may occur, in general, counseling is provided in the following manner. Fetal echocardiographic imaging is performed in a dedicated fetal heart imaging suite, located in a separate facility from the pediatric echocardiography laboratory. In parallel, obstetrical anatomical scan imaging is performed and evaluation offered by a dedicated group of maternal–fetal medicine specialist at CHOP. Following imaging, families are brought into a private consultation room, where findings and counseling by a cardiologist, nurse, and social worker are offered as a team, with cardiologist taking the lead in conversation. The encounter includes face-to-face review of patient history, explanation of normal heart structure and function, and conveyance of how current findings of the cardiac anatomy on fetal echocardiography differ from normal. Visual tools such as diagrams of normal and abnormal cardiac structure, either pre-formatted or drawn uniquely for the condition present, are used to aid in explanation. Counseling includes discussion of impact on delivery plans, postnatal treatment course, surgical options as well as short- and long-term outcomes. Further education, family assessment, and ongoing concerns are addressed by the fetal heart nurse and social worker. Following initial encounter, additional visits occur serially every 4 weeks until delivery, with updated fetal echocardiographic imaging and repeat counseling sessions to supplement and review findings and answer questions. Fetal heart nurse coordinators are in frequent touch with families in response to queries that arise between visits, through phone calls or email communications. Towards the end of gestation, families have an opportunity for a single encounter meeting with a heart surgeon, accompanied by the fetal heart nurse. A tour of the cardiac intensive care unit is offered.

### Survey Protocol

An online survey created in REDcap (research electronic data capture) was distributed to family email addresses recorded in the electronic health record (Epic). The survey consisted of basic demographic information, Likert scales (grade 1–5, with 1 as lowest and 5 as highest score) assessing parental grade with respect to various aspects of prenatal counseling, and open-ended questions to elicit further free text response (Table 1, survey). Survey questions solicited grades from parents concerning various aspects of prenatal counseling and were developed specifically for this study, based on feedback during discussion with families. The first question related to grading the recollection of prenatal counseling. This was followed by requests for assessments of the following: (1) overall impression of quantity of counseling, (2) explanation of the heart defect, (3) preparation for heart surgery, (4) preparation for the hospital course and care, (5) preparation for complications and outcomes of a Fontan circulation, and (6) preparation for neurological, school-related, or behavioral problems. Survey questions then solicited impressions concerning how helpful it was to meet with specific FHP members including (1) fetal cardiologist, (2) fetal heart nurse, (3) fetal heart social worker, and (4) cardiac surgeon.

**Table 1:** Fetal counseling survey questions administered through REDcap to all participating families

Demographics
1. Parent respondent (mother or father)
2. My child was born in the CHOP special delivery unit (yes or no)
3. Delivery type (vaginal vs c-section)
4. To the best of my memory, the number of days my baby was hospitalized following birth until discharge to home was:
5. Child's current age

Counseling satisfaction and preparation	Choose one
6. When I think about my prenatal fetal heart counseling…	(1) I have no or only very few rare memories of my experiences
	(2) I have some memory of my experiences
	(3) I have a fair memory of my experiences
	(4) I have a good memory of my experiences
	(5) I have excellent, vivid memories of my experiences
7. When I think about the number of prenatal fetal heart counseling sessions, I feel that…	(1) I did not receive an appropriate number of sessions
	(2) I received a few sessions but should certainly have had more
	(3) I received a fair number of sessions
	(4) I received a very good and appropriate number of sessions
	(5) I received an excellent, great number of sessions
8. When I think about my prenatal fetal heart counseling and the explanation of the heart condition, I feel that…	(1) I did not receive a good explanation of the heart condition and did not understand my baby's problem at birth
	(2) I received some of an explanation of the heart condition, but still found it difficult to understand my baby's problem at birth
	(3) I received a fair explanation and understood aspects of my baby's problem at birth
	(4) I received a very good explanation and had a good understanding of my baby's problem at birth
	(5) I received an excellent explanation and had an excellent understanding of my baby's problem at birth
9. How well do you feel the prenatal counseling that you received prepared you for what to expect about…the surgery?	(1) Not at all prepared (2) Somewhat or minimally prepared (3) Fairly prepared (4) Very well prepared (5) Extremely well prepared
10. How well do you feel the prenatal counseling you received prepared you for what to expect about…the hospital course and care?	
11. How well do you feel the prenatal counseling you received prepared you for what to expect about…complications and outcomes of a Fontan circulation?	
12. How well do you feel the prenatal counseling you received prepared you for what to expect about…neurological, school-related, or behavioral problems?	

Provider team assessments	
13. In my prenatal fetal heart counseling, I found meeting with the fetal cardiologist:	(1) Not helpful at all (2) Somewhat or minimally helpful (3) Fairly helpful (4) Very helpful (5) Extremely helpful (6) Did not meet
14. In my prenatal fetal heart counseling, I found meeting with the fetal heart nurse:	
15. In my prenatal fetal heart counseling, I found meeting with the fetal heart social worker:	
16. In my prenatal fetal heart counseling, I found meeting with the cardiac surgeon:	

Only one parent was required to respond and discussion between parents was allowed. The questionnaire was distributed via email to potential candidates 3 times over the course of 2 months to increase response rates. Responding families were assigned a study ID for deidentification purposes. Once a response cohort was identified, chart review was performed to examine patient medical records in a manner blinded to specific survey responses. Data collection included condition variables as well as factors that influence magnitude of burden of care and potentially impact perceptions of prenatal counseling: cardiac diagnosis, length of hospital stays for all stages of surgical palliation, type of Fontan, utilization of ECMO, number of cardiac catheterizations, additional surgeries beyond anticipated, other non-cardiac diagnoses, use of nasogastric feeding tube, and current medication regimen.

### Data Analysis

Data analysis consisted of creating scores for each Likert scale variable by averaging the numerical ratings (1–5) for the survey response cohort. Paired t-tests were used to explore for significant differences between the survey variables of various aspects of prenatal counseling, and between ratings for FHP counseling team members. A composite impression of overall quality of counseling—a “comprehensive fetal counseling score (FCS)”—was calculated for each participant by averaging their individual Likert scale ratings across all 10 survey questions including those concerning prenatal counseling content (6 questions) and the provider assessments (4 questions).

To explore the influence of postnatal course and disease severity on current impressions of prenatal counseling, the magnitude of burden of disease was assessed and compared across the survey responses. Chart review data analysis consisted of determining 5 indicators of overall burden of disease. These included (1) parental report of their estimate of number of days of initial newborn hospitalization after birth for first stage of care, (2) discharge to home after initial hospitalization with or without a feeding tube, (3) current cardiac medication burden as a reflection of magnitude of care, dichotomized into 2 groups: (a) those who only received aspirin alone or aspirin and enalapril (standard medications) and (b) those who received additional medications, (4) presence of other comorbid conditions or complications, and (5) unexpected interventions such as unanticipated surgeries or cardiac catheterizations. Parental report of days of initial newborn hospitalization was compared with actual medical record recording.

In order to test for possible associations between burden of disease severity and perception of fetal counseling, non-parametric independent *t*-tests (Independent-Samples, Two-Tailed Mann–Whitney *U* Test) were used to determine if there were significant differences in composite FCS values between (1) those with and without unexpected cardiac surgeries, (2) those with and without unexpected catheterizations, (3) those with and without comorbid conditions, (4) those who were and were not discharged home with a feeding tube, (5) those who had a standard (aspirin with or without enalapril) or increased (more than aspirin and enalapril) medication regimen, and (6) those who received or did not receive extracorporeal membrane oxygenator (ECMO) support at any point during care. Pearson correlation test was used to determine the association between hospitalization length and comprehensive fetal counseling score. A *p* value of < 0.05 was considered significant.

## Results

### Subject Characteristics

In total, 248 families with prenatal diagnosis of single ventricle congenital heart disease meeting study criteria were identified. Of these, 65 responded to the questionnaire (26% response rate) with 6 responding to only a very limited number of survey questions, hence the cohort for complete analysis consisted of 59 subjects. Respondents included 62 mothers (95%) and 3 fathers (5%). Of the overall respondents, 62 (95%) delivered at the CHOP Special Delivery Unit and 3 (5%) delivered elsewhere. Deliveries consisted of 40 (62%) vaginal births and 25 (39%) cesarian section deliveries. Of the 59 respondents with completed surveys, 11 (19%) had a fetus with dominant single left ventricle anatomy and 48 (81%) had a dominant single right ventricle anatomy. With regard to initial palliation, 22 (37%) received the Norwood procedure with a Blalock–Taussig shunt, 29 (49%) received the Norwood procedure with a Sano shunt, 5 (9%) underwent pulmonary arterial banding, 1 (2%) received a ductus arteriosus stent, and 2 (3%) received no initial intervention. Prenatal counseling consisted of 3–5 in-person encounters, with fetal heart nurse coordinator follow-up via email and phone calls throughout the prenatal period. At the time of survey administration, 38 patients (64%) completed the final stage of Fontan palliation; 29 (76%) with extra-cardiac fenestrated conduit; and 9 (24%) a lateral tunnel with fenestration. Average age of the children at the time of parental survey was 4.6 ± 3.3 years (range 1–10 years).

### Survey Scores

**Fig. 1:**
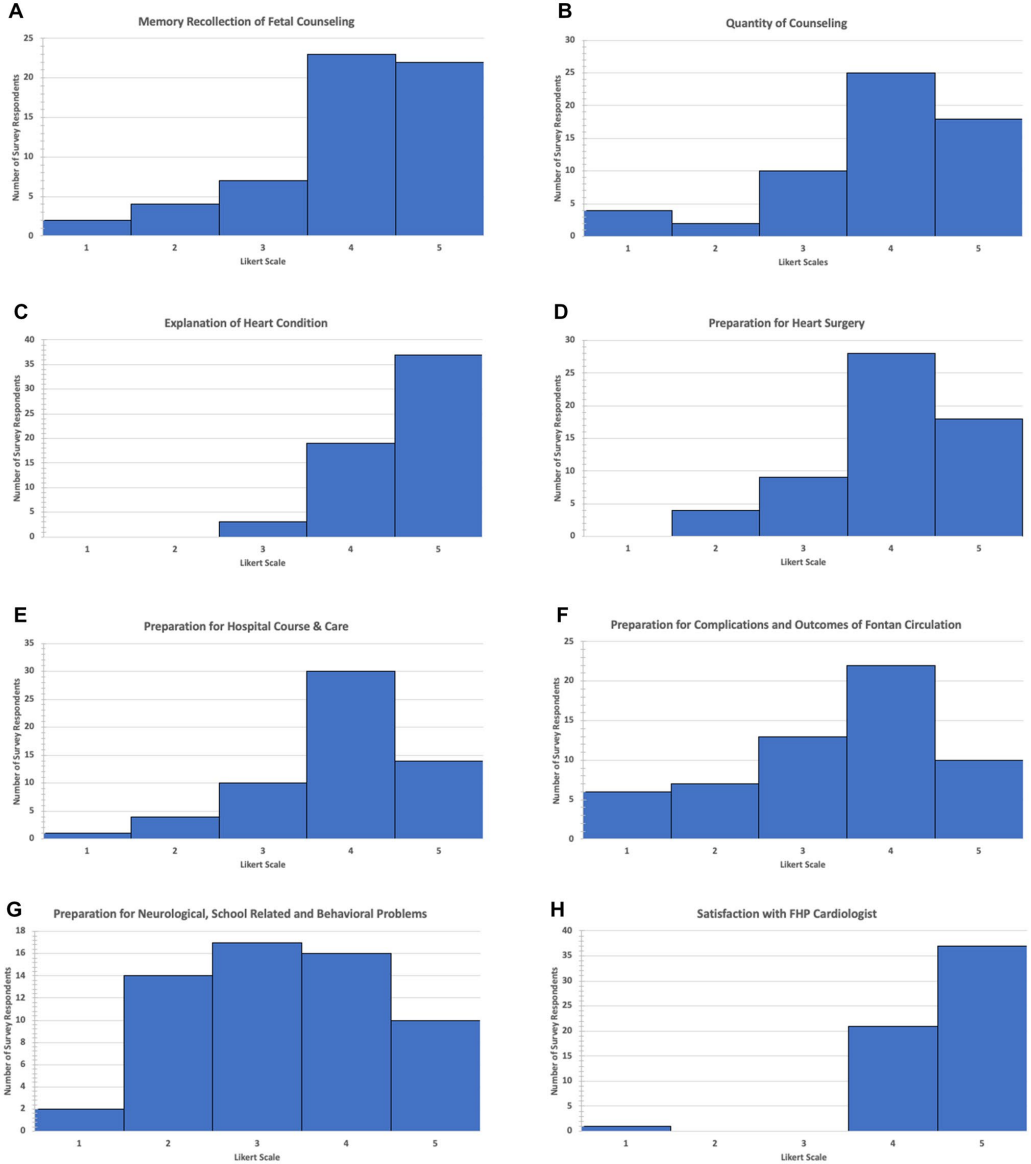

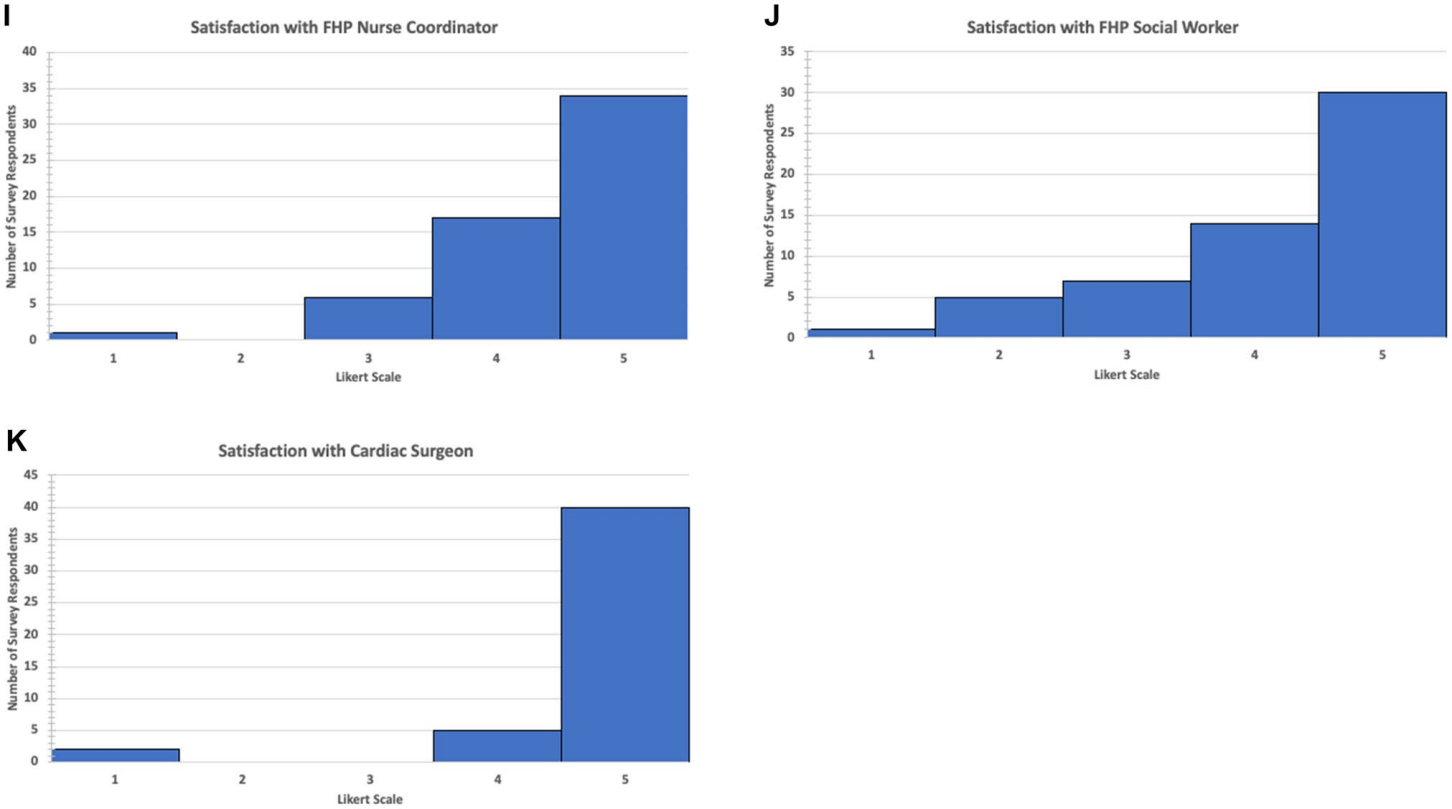
Distribution of survey scores for domains of counseling, FHP provider satisfaction scores, and composite comprehensive fetal counseling score. Graph displays number of survey respondents for each grade 1–5 for survey questions related to **A** explanation of heart condition, **B** quantity of counseling, **C** memory recollection of fetal counseling, **D** preparation for heart surgery, **E** preparation for hospital course and care, **F** preparation for complications and outcomes of Fontan circulation, **G** preparation for neurological, school-related, and behavioral problems, **H** satisfaction with the fetal heart program (FHP) cardiologist, **I** satisfaction with the FHP nurse coordinator, **J** satisfaction with the FHP social worker, and **K** satisfaction with cardiac surgeon

Table 2 shows the mean ± SD values for the survey variables and Fig. 1a–k shows the distribution of survey scores for domains of counseling, FHP provider satisfaction scores, and composite comprehensive fetal counseling score. Memory recollection of fetal counseling scores were generally quite high with no association to the comprehensive fetal counseling score. With regard to preparation for various aspects of care, average scores were highest for explanation of heart condition and lowest for preparation for neurological, school, and behavioral issues. The average level of preparation for complications and outcomes of the Fontan circulation was significantly lower than preparation for surgery (*p* < 0.01) and hospital course (*p* < 0.05). The average level of preparation for neurological, school, and behavioral issues was significantly lower than surgery (*p* < 0.001) and hospital course (*p* < 0.001).

**Table 2:** Mean (SD) values for the individual survey variables of counseling content, provider satisfaction scores, and composite comprehensive fetal counseling score

Variable	Survey scores
Overall memory recollection of prenatal counseling	4.0 (1.1)
*Specific elements of counseling*	
Quantity of counseling	3.9 (1.1)
Explanation of heart condition	4.6 (0.6)
Preparation for heart surgery	4.0 (0.9)
Preparation for hospital course and care	3.9 (0.9)
Preparation for complications and outcomes of Fontan circulation	3.4 (1.2)
Preparation for neurological, school-related, and behavioral problems	3.3 (1.1)
*Individual providers of the fetal heart counseling team*	
Fetal Cardiologist	4.6 (0.7)
FHP Nurse Coordinator	4.4 (0.8)
FHP Social Worker	4.2 (1.1)
Cardiac Surgeon	4.7 (0.9)
Composite fetal counseling score (FCS)	4.1 (0.7)

With regard to satisfaction with various members of the FHP provider team, cardiac surgeon had the highest average score although 12 (20%) families did not meet this team member before birth. Social worker had the lowest average score. When comparing team member scores among the 45 families who met with all providers, the average social worker score was significantly lower than the cardiologist (*p* < 0.05) and surgeon (*p* < 0.05).

### Burden of Disease Impact on Perceptions of Prenatal Counseling

**Fig. 2:**
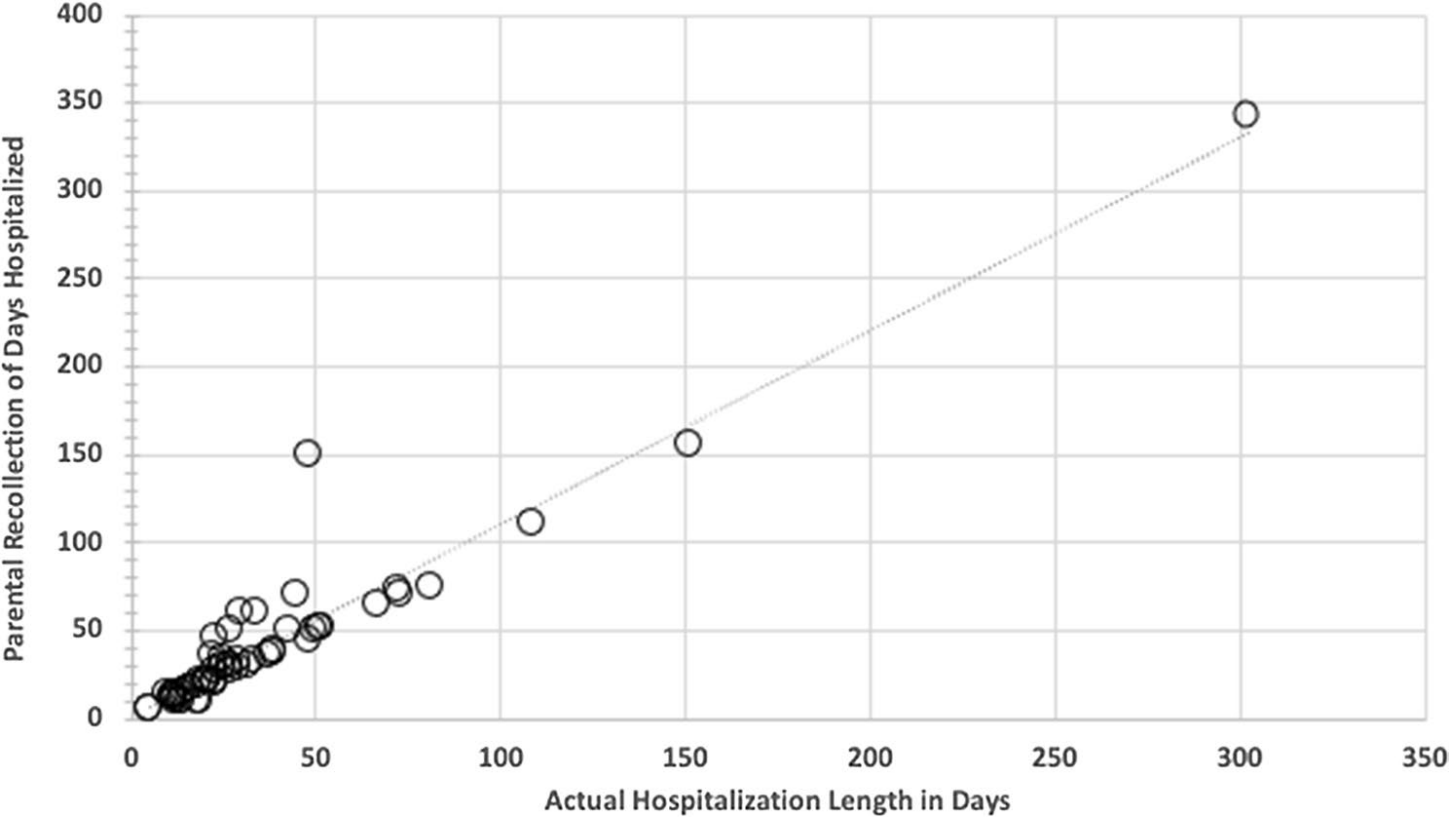
Parental recollection of initial newborn hospitalization length of stay versus actual measure of days of hospitalization from the medical record (Pearson *r* = 0.953, *p* < 0.001)

**Fig. 3:**
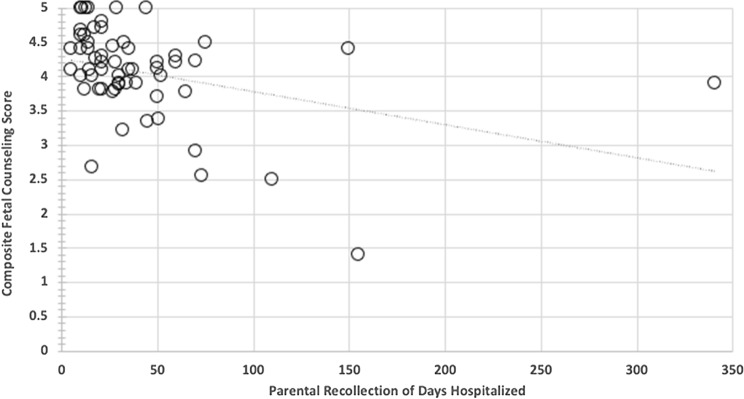
Statistically significant negative correlation between the composite fetal counseling score and parental recollection of length of hospitalization (Pearson *r* = − 0.357 *p* < 0.01)

The average estimated days of hospitalization after birth by parental report is 36 ± 31 days with a range of 5–150 (median 28) days, with one outlier of 341 days. Actual newborn hospitalization was 32 ± 26 days with a range of 5–146 (median 24) days excluding one outlier of 302 days. Parental recollection of initial newborn hospitalization length of stay matched very well to the actual measure of days of hospitalization from the medical record (Fig. 2, Pearson *r* = 0.953, *p* < 0.001). A statistically significant negative correlation was found between the composite FCS and parental recollection of length of hospitalization (Fig. 3, Pearson *r* = − 0.357 *p* < 0.01).

Table 3 lists the distribution of elements of disease burden assessed in the survey study cohort. The composite FCS for the entire study cohort was a mean of 4.1 ± 0.7. No statistically significant differences were found in the composite FCS between those with or without a feeding tube, those with differing medication regimens, those with additional comorbidities or complications, and those who required unexpected surgeries or catheterizations. However, the composite FCS value was significantly lower for those who had ECMO care versus those who did not (avg 3.3 ± 1.1 vs 4.2 ± 0.5, *p* = 0.047).

**Table 3:** Burden of disease experienced by the survey study cohort (*n* = 59)

Variable	No (% of cohort)
Discharged to home as a newborn with feeding tube	40 (68%)
Current medication burden beyond aspirin alone or aspirin and enalapril	29 (49%)
Comorbidities and complications	21 (36%)
Unanticipated additional cardiac surgery	8 (14%)
Unanticipated cardiac catheterization	26 (44%)
ECMO	7 (10%)

## Discussion

In this report, we describe feedback from families on the efficacy of prenatal counseling for SVCHD. Despite its importance and value, judging the adequacy of quantity and quality of counseling at the time of prenatal care is difficult, as there are no applicable tools or counseling specific metrics available. Gauging the effectiveness of prenatal counseling through survey of families who have lived through the early experiences of care for SVCHD as well as assessing the impact of modifiers on perception of counseling such as burden of disease can provide feedback for areas of strength and deficiencies as providers help families prepare for this complex condition [7].

Overall, we found that counseling is perceived favorably upon reflection back by a cohort of families with prenatal diagnosis of SVCHD. Survey scores in general were high for a variety of elements of counseling. Scores were highest for explanation of the heart condition, and lowest for preparation concerning neurological, school-related, and behavioral problems. These findings are interesting and revelatory. Of all the information necessary to convey, the nature of the cardiac condition, no matter how complex, is probably the most concrete to communicate. A systematic approach of first explaining the normal, and then pointing out the abnormal heart, is commonly deployed. Various aids such as diagrams and videos are available as resources for reinforcement, with information dictated reliably as the counselor adheres to the findings of the echocardiographic examination. Due to the concrete nature of this content, providers are likely more comfortable and thus more effective in communicating it to families. On the other hand, while abundant data now readily indicate the presence of neurological, school-related, and behavioral problems in many with SVCHD [8], the prevalence of the problem and depth of severity is highly variable in this population. There is little prenatal data that can accurately predict outcome [9], thus prenatal counseling involves communication of risk, not certainty [10]. Such concepts can be a challenge to effectively convey to families without causing distress, as perhaps only second to survival, neurological outcomes are appropriately high on the mind of concerned parents. Prenatal providers may also not be as familiar with neurological outcomes data [11] and thus not as comfortable in counseling and answering questions on this topic, to the same extent as questions related directly to the heart.

Scores for all members of the fetal heart counseling team were high, with the cardiac surgeon scoring highest and the social worker scoring lowest within the group. These differential provider score findings on the surface were somewhat surprising, but perhaps understandable, with the following plausible explanations. In our practice, encounter with the cardiac surgeon occurs at the near end of gestation with the purpose of engagement to meet the provider who will be performing the operation and as a representative, lead member of the postnatal care team. By this time, the bulk of prenatal counseling information concerning type of heart condition, surgeries required, as well as discussion of prognosis and outcomes will have been delivered by the cardiologist and nurse coordinators. The cardiac surgeon provides an overall summary and an opportunity for any questions to be answered concerning the operative details and other surgical matters. Families very much value this engagement, which in our unique practice, functions as a final confirmatory coda to the prenatal counseling content previously received. Meeting the surgeon provides confidence to families and sends the message of a seamless care plan with readiness for transition to newborn management. On the other hand, social worker encounters occur multiple times throughout the prenatal period, and often relate to psychosocial assessments with management of parental stress. Satisfaction with the social worker may also reflect adequacy of receipt of services such as travel, lodging, childcare, and other logistics related to delivery plans, many of which can be challenging to achieve in a satisfying manner. Although of great help and relief for many families, social service care deficiencies may be tagged to the social worker and thus recollections attributed. Importantly, despite the statistically significant differences in some of the provider scores, they are not clinically meaningful and overall satisfaction with all providers was very high. The multidisciplinary aspect of prenatal counseling remains an essential approach and is the ideal structure in our view.

We were curious to see if postnatal outcomes influenced the perceptions of prenatal counseling. We found that overall perceptions of prenatal counseling for SVCHD are indeed influenced by severity of disease burden as reflected by initial newborn length of hospital stay. First, it is interesting to note that parental recollection of length of stay for initial hospitalization was highly accurate and correlated very well with actual medical record days of initial hospitalization. Furthermore, longer length of stay was associated with poorer overall comprehensive fetal counseling scores. Measures of disease burden specific to this population such as utilization of feeding tube, comorbidities, and complications, additional procedures such as unanticipated catheterization or surgeries, and medication regimen were not associated with fetal counseling scores. However, utilization of ECMO was significantly associated with poorer fetal counseling scores. Length of stay and utilization of ECMO are highly associated co-variates [12]. This suggests that dramatic, undesirable elements of the postnatal course such as use of ECMO and a lengthy hospital stay may influence recollection of the quality of prenatal counseling, or that perhaps our prenatal counseling in fact, did not adequately prepare families for these possibilities.

Our study has several limitations. The patient cohort was a relatively small percentage (26%) of all possible candidates. As in all voluntary survey type studies, there is the likelihood of selection bias in the group of respondents. Social, demographic, and educational levels of families certainly influence the survey responses. Contact information was pulled from the EPIC record, which may not have been updated since initial entry and the survey was only offered in English. Although patients with major extra-cardiac anomalies were excluded, associated anomalies such as genetic abnormalities were not excluded and may have contributed to parental responses. By nature of the study, we only surveyed families who chose to proceed with care and in which patients have survived. Of great interest, but not addressed in this investigation, would be to solicit feedback on prenatal counseling from families in whom there has been a loss or in those who may have chosen termination of pregnancy. As there are no standards or established protocols for prenatal counseling for SVCHD, content and style may vary from practice to practice. Our findings may therefore not be transferable to experiences at other institutions, for example at centers where cardiac surgeons participate in early fetal counseling sessions.

Our report describes feedback via survey on perceptions of prenatal counseling for SVCHD. What have we learned? By and large, our prenatal counseling is perceived favorably, but there are areas for improvement. Counseling concerning neurological, school-related, and behavioral problems can be improved. Properly informing about the risks for lengthy hospitalization and possible need for ECMO are required. Doing so in a manner that describes the risk of these outcomes, yet places the likelihood in perspective, is necessary, with a delivery that is balanced between reality and hopeful optimism. This can be a challenge but should be approached and not shied away from by providers. Establishment of protocols and creating checklists of content for prenatal counseling will aid providers by creating structure to these sessions [13]. Training prenatal counselors with observed mock counseling sessions and providing feedback will raise the skill level and comfort of providers and should be initiated as part of medical education for those who will offer these services. Further studies analyzing prenatal counseling techniques and content can help improve upon the delivery of this important aspect of prenatal care [14].
